# Health literacy in patients participating in cardiac rehabilitation: A prospective cohort study with pre-post-test design

**DOI:** 10.1016/j.ijcrp.2024.200314

**Published:** 2024-07-22

**Authors:** Pernille Lunde, Jostein Grimsmo, Birgitta Blakstad Nilsson, Asta Bye, Hanne Søberg Finbråten

**Affiliations:** a Department of Rehabilitation Science and Health Technology, Oslo Metropolitan University, PB 4, St.Olavs plass, 0130, Oslo, Oslo, Norway; b Department of Cardiac and pulmonary Rehabilitation, Cathinka Guldberg′s Hospital, Ragnar Strøms veg 10, 2067, Jessheim, Jessheim, Norway; c Section for Physiotherapy, Oslo University Hospital, PB 4950 Nydalen, 0424, Oslo, Oslo, Norway; d Department of Nursing and Health Promotion, Oslo Metropolitan University, PB 4 St.Olavs plass, 0130, Oslo, Oslo, Norway; e European Palliative Care Research Centre (PR China), Department of Oncology, Oslo University Hospital and University of Oslo, PB 4950 Nydalen, 0424, Oslo, Oslo, Norway; f Department of Health and Nursing Sciences, Inland Norway University of Applied Sciences, PB 400, N-2418, Elverum, Elverum, Norway

**Keywords:** Health literacy, Cardiac rehabilitation, Secondary prevention

## Abstract

**Background and aims:**

Adherence to recommendations regarding medical treatment and healthy behaviour serve as a significant challenge for patients experiencing a cardiac event. Optimizing the patients’ health literacy (HL) may be crucial to meet this challenge and has gained increased focus the last decade. Despite cardiac rehabilitation (CR) being a central part of the treatment of patients experiencing a cardiac event, such programs have not been evaluated regarding HL. Therefore, the aim of this study was to describe and evaluate HL in patients participating in CR.

**Methods:**

A prospective cohort study with pre-post-test design of patients participating in CR. Data were collected at program admission and completion (August 2017–June 2018). Patients from three different CR-programs were included. Descriptive and inferential statistics were applied to describe and evaluate HL and change in HL across categories of demographical variables and type of rehabilitation.

**Results:**

In total, 113 patients attending CR were included. A statistically significant increase in HL was observed from pre-to post-CR (mean change: 2.24 ± 3.68 (*p* < 0.001)). Patients attending 12-weeks outpatients CR-program had statistically significant higher HL, both at pre- and post-CR, compared to those attending one-week residential CR.

**Conclusions:**

Participation in CR statistically significantly improves HL. Overall, judging health information was found as the most difficult aspect of HL, both at pre- and post-CR. This should be emphasized in secondary prevention to overcome barriers related to adherence to medical treatment and healthy behaviour.

## 1 Introduction

Adherence to recommendations regarding medical treatment and healthy behaviour is crucial to improve long-term prognosis after cardiovascular diseases (CVDs), such as coronary artery disease (CAD) [1,2]. Today, adherence presents a significant challenge, resulting in few patients achieving the guideline standard for secondary prevention years after experiencing a cardiac event [3]. Recently, the European Association of Preventive Cardiology published a clinical consensus statement on how to optimize the adherence to a guideline directed medical therapy in secondary prevention of CVDs [4]. According to this statement, optimizing the patients’ health literacy (HL) is crucial in promoting adherence in secondary prevention [4]. Health literacy originated in the mid-1970s, and since then, HL has been defined in several ways. Based on a systematic literature review, Sørensen et al. [5] not only developed an integrative model for HL, but also defined HL as following:

‘*people’s knowledge, motivation and competences to access, understand, appraise and apply health information in order to make judgments and take decisions in everyday life concerning health care, disease prevention and health promotion to maintain or improve quality of life during the life course*‘ [[5], p. 3].

Health literacy could be considered as a prerequisite for being able to deal with health information and convert the health information into health promoting behaviour.

Large proportions of individuals with CVD, or receiving preventive treatment for CVD could be considered having low HL [6,7]. Low levels of HL in patients with CVD is associated with lower disease-related knowledge, less blood pressure control, less self-management behaviours, such as exercise behaviours and bodyweight-monitoring, as well as reduced quality of life [8,9]. Moreover, low HL is associated with increased risk for readmission and increased risk of mortality [10]. Individuals with low HL could be positive to consider preventive lifestyle changes, but might struggle to initiate such changes [7]. Consequently, HL could be deemed as an important determinant in whether a patient is able to initiate and maintain healthy behaviour [11]. Considering the patients’ HL and tailoring health information accordingly is important to enhance adherence and optimize health outcomes.

The beneficial effects of cardiac rehabilitation (CR) are well demonstrated and currently CR has a class IA recommendation in the European guidelines on CVD prevention [1,12]. Cardiac rehabilitation is a comprehensive programme that involves exercise training, risk factor modification, education and psychological support [1]. In this, education including comprehensible information on perception of disease, empowerment and self-management as well as information and motivation on target lifestyle modifications and pharmacological treatment target are central [1]. Prior to providing education, the importance of evaluating literacy level is emphasized [1]. Further, strategies that address barriers to improving modifiable risk factors due to varying HL levels are crucial for reducing the risk of future CAD events [13]. The delivery of CR should be personalized to provide support and education to enhance the patients HL abilities. This could improve the long-term prognosis of patients participating in CR.

Despite the emphasis on HL in the recently published clinical consensus statement on how to optimize the adherence in the secondary prevention of CVDs [4], no studies in Europe have evaluate how participation in CR influence HL. Therefore, the primary aim of this study was to describe and evaluate HL in patients participating in CR. Additionally, differences in HL in the CR population regarding age, sex, educational level, and type of CR program attended, were investigated.

## 2 Methods

### 2.1 Study design

This study was a prospective cohort study with pre-post-test design of patients participating in CR. The study was a sub study of a larger project in which was approved by the Regional Committee for Medical and Health Research Ethics (South-East ID: 2016-1476). The study was conducted according to the Helsinki Declaration. All patients provided informed, written consent.

### 2.2 Setting

Patients were recruited from two CR centers in the eastern part of Norway where in total three CR programs were offered: 12-week outpatient CR, four-week inpatient CR and one-week inpatient CR (presented below). Physicians referred patients to these CR-programs after the patient were medical stable or after various cardiac diseases. Patients were referred to CR-programs primarily based on the referral physicians’ preferences and knowledge, and geography.

Cardiac rehabilitation programs.

The 12-weeks outpatient CR-program was an exercise-based CR-program where the patients spend two to 3 h, two to three times a week for 12 weeks at the CR-centre. In addition to exercise and group-based teaching sessions, individualized follow-up was provided depending on each patient needs, in example, optimalization of medication, psychology consulting, dietary guidance or support for smoking cessation. The 12-week outpatient CR-program have previously been described and evaluated regarding exercise capacity and quality of life [14]. While the one-week residential CR program aimed to assess, motivate and start or continue a lifestyle change for patients with established cardiac diseases, similar as reported by Bergum and colleagues [15], the four-week residential CR-program was laid out in the same way. However, the patients obviously had longer time to initiate the lifestyle changes and to increase their knowledge related to their disease and health and has previously been reported as usual care [16]. Healthcare professions involved in CR-programs included cardiologist, physiotherapist, dietician, psychologist. In addition, nurses were involved in the residential CR-programs.

When completing the CR-programs, patients were encouraged to adhere to treatment and healthy behaviour, and further to schedule appointments with their general practitioner and/or physiotherapist as necessary.

### 2.3 Participants

Patients were included in the present study if they were eligible and included to the main project [17] of which the present study was a sub study of. The inclusion criteria were the following: patients completing CR at one of the three CR-programs; age ≥40 years; owner and user of an Android or Apple smartphone; and able to read and understand Norwegian or English. The exclusion criteria included ischemia or arrhythmias uncovered at cardiopulmonary exercise test (CPET) that gave restrictions equivalent to <80 % of maximal heart rate or BORG scale (6–20) <15 at exercise. Patients with muscular or skeletal disorders that affected exercise capacity more than the cardiac disease were also excluded. Additionally, patients with severe malignant disease that affected the patient’s life span to a greater extent than their cardiac disease were also excluded.

### 2.4 Assessments

Data collection was conducted at program admission and completion (August 2017–June 2018). All patients were assessed at entry to the CR-program (baseline, pre-CR) and at completion of the CR-program (post-CR). At baseline, data regarding age, sex and educational levels i.e. number of years of education beyond upper secondary school, were collected. They also completed the HL questionnaire, both at pre- and post-CR. Based on sample size calculation, for the project of which the present study was a sub-study of, we aimed to include 113 patients in total [17].

### 2.5 Health literacy

The Health Literacy Survey 12 questions (HLS-Q12) [18], a short version of the European Health Literacy Survey Questionnaire [19], was used to measure HL. HLS-Q12 reflects the conceptual model of HL developed by Sørensen et al. [5], and measures HL proficiency across four cognitive domains (access, understand, appraise and apply health information) and three health domains (health care, disease prevention and health promotion). By combining the four cognitive domains with the three health domains, a 4 x 3 cell HL matrix is constituted. The HLS-Q12 consists of 12 items, where each cell of the matrix is represented by one item. The HLS-Q12 has previously been validated in people with type 2 diabetes [20] and in the general Norwegian population [18]. The validated version of HLS-Q12 offers four response categories (1) very difficult; 2) difficult; 3) easy and 4) very easy), where higher scores indicate higher HL proficiencies. The version used in the present study offers six response categories from very difficult (1) to very easy (6), where only the extreme categories were labelled. Hence, assessing psychometric properties of this version was considered necessary before evaluating HL in the CR population.

#### 2.5.1 Psychometric properties of the HLS-Q12

When testing HLS-Q12 data against the partial credit parametrisation [21] of the unidimensional Rasch model [22], three items (4, 5 and 9) displayed unordered response categories when six response categories was applied. Hence, we rescored the response categories by collapsing the response categories 2 and 3, and 4 and 5 (keeping the extreme categories). Applying four response categories for all 12 items, the HLS-Q12 data fit the unidimensional Rasch model (having a non-significant total-item chi square) and displayed relatively high reliability indexes (Person Separation Index: 0.828, Cronbach’s alpha: 0.828). The scale could be considered sufficiently unidimensional as the proportions of individuals with significantly different person-location estimates on a pair of compared subscales was 7.34 % with a lower 95 % confidence interval (CI) proportion of 0.032 (the proportion of individuals with significantly different person-location estimates on the pair of compared subscales should be lower than 5 % (or the lower bound of the binominal 95 % CI should be below 0.05) [23]). As well-targeted scales should have a mean person location value around zero [24], the targeting of the scale could have been better (mean persons location estimate: 1.836). Chi-square statistics and standardised residuals based on comparisons between observed and expected values were used to assess item fit. Chi-square probability values above Bonferroni’s adjusted 5 % and fit residuals in the range ±2.5 indicate adequate item fit [24]. All items displayed acceptable fit to the Rasch model and discriminated well (item fit residuals between −1.098 and 1.173). Applying two-way analysis of variance of standardised residuals [23], none of the items displayed statistically significant differential item functioning (DIF) across categories of the characteristics sex, age, education, or type of rehabilitation, indicating that the items work invariantly for the available sociodemographic variables. Statistical significance was assumed at a Bonferroni-adjusted 5 %. The software RUMM2030Plus [25] was applied for Rasch analysis.

### 2.6 Statistical analyses

As the HLS-Q12 items with four response categories fit the unidimensional Rasch model we added item scores into a total score of HL yielding a possible score of 12–48. The HL total score was considered normally distributed. Paired samples *t*-test was performed to investigate the change in HL score from pre-to post-test. Independent samples *t*-tests and one-way analysis of variance (ANOVA) were applied to study differences in the change of HL score across categories of sociodemographic variables (age, sex, educational level) and type of CR program attended.

Independent samples *t*-tests were conducted to study differences in HL score across categories of sex and age (dichotomized around mean). When studying differences in HL score across years of education and the three types of rehabilitation, ANOVA was used. Statistical analysis was performed using the Statistical Package for Social Sciences (SPSS), version 28 (IBM Corporation). Statistical significance was assumed at p < .05 (two-tailed).

## 3 Results

In total, 113 patients were included and are presented in Table 1. The age of the patients ranged from 40 to 77, and 78 % were males. Approximately 40 % had education of four to twelve years beyond upper secondary school.

**Table 1:** Baseline characteristics (n = 113).

Variable	Mean ± sd/number (%)
**Males**	84 (78)
**Age**	59 ± 9
**Body mass index**	29.2 ± 4.9
**Bodyweight (kg)**	90.7 ± 16.8
**Blood pressure (mmHg)**	
**Systolic**	135 ± 17
**Diastolic**	82 ± 10
**Current smoker**	4 (3.5)
**Education**	2.9 ± 2.7
**Diagnose**	
**Coronary artery disease**	83 (73.4)
**Valve**	19 (16.8)
**Other**	11 (9.8)
**Treatment**	
**Percutaneous coronary intervention**	55 (48.7)
**Coronary artery bypass graft**	22 (19.5)
**Valve surgery**	19 (16.8)
**Conservatively**	10 (8.8)
**Other**	7 (6.2)
**Medication**	
**Beta-blocker**	69 (61.1)
**Statins**	96 (85)
**ASA + plate inhibitor**	75 (66.4)
**Antihypertensive**	55 (48.7)
**Peak oxygen uptake (ml/kg/min)**	28.1 ± 6.9
**Type of rehabilitation**	
**1 week residential**	35 (31)
**4 weeks residential**	40 (35.4)
**12 weeks outpatient**	38 (33.6)

ASA: acetylsalicylic acid; sd; standard deviation; Education: years beyond upper secondary school.

Both at pre- and post-CR, the most difficult HL tasks were to judge the advantages and disadvantages of different treatment options (item 3), to judge if the information on health risks provided by the mass media is reliable (item 7) and to decide how one can protect oneself from illness based on advice from family and friends (item 8; Table 2). At pre-CR about 40 % found these tasks difficult, whereas 22, 29 and 25 %, respectively, found these tasks difficult post-CR. Four patients were not offered HLS-Q12 at pre-CR. Consequently, 109 patients were included at pre-CR.

**Table 2:** Category frequencies, n (%), for each of the HLS-Q12 items at pre- and post-CR, respectively.

Item no	**HLS**_**19**_ **HLS-EU** **Item no**	**How easy would you say it is**	Pre-CR, n = 109				Post-CR, n = 113			
			**Very difficult**	**Difficult**	**Easy**	**Very easy**	**Very difficult**	**Difficult**	**Easy**	**Very easy**
1	CORE-HL2	… to find information on treatments of illnesses that concern you?	2 (1.8)	17 (15.6)	74 (67.9)	16 (14.7)	0 (0)	14 (12.4)	66 (58.4)	33 (29.2)
2	CORE-HL7	… to understand what to do in a medical emergency?	0 (0)	23 (21.1)	70 (64.2)	16 (14.7)	0 (0)	14 (12.4)	76 (67.3)	23 (20.4)
3	CORE-HL10	… to judge the advantages and disadvantages of different treatment options?	2 (1.8)	41 (37.6)	63 (57.8)	3 (2.8)	1 (0.9)	24 (21.2)	84 (74.3)	4 (3.5)
4	CORE-HL14	… to follow instructions on medication?	0 (0)	11 (10.1)	52 (47.7)	46 (42.2)	1 (0.9)	4 (3.5)	46 (40.7)	62 (54.9)
5	CORE-HL18	… to find information on how to handle mental health problems like stress or depression?	3 (2.8)	34 (31.1)	65 (59.6)	7 (6.4)	0 (0)	20 (17.7)	72 (63.7)	21 (18.6)
6	CORE-HL23	… to understand why you need health screenings?	0 (0)	4 (3.7)	46 (42.2)	59 (54.1)	1 (0.9)	2 (1.8)	47 (41.6)	63 (55.8)
7	CORE-HL28	… to judge if the information on health risks in the mass media is reliable?	4 (3.7)	39 (35.8)	58 (53.2)	8 (7.3)	3 (2.7)	30 (26.6)	67 (59.3)	13 (11.5)
8	CORE-HL30	… to decide how you can protect yourself from illness based on advice from family and friends?	5 (4.6)	39 (35.8)	59 (54.1)	6 (5.5)	2 (1.8)	26 (23.0)	74 (65.5)	11 (9.7)
9	CORE-HL32	… to find information on healthy life styles such as physical exercise, healthy food and nutrition?	0 (0)	12 (11.0)	68 (62.4)	29 (26.6)	1 (0.9)	1 (0.9)	68 (60.2)	43 (38.1)
10	CORE-HL38	… to understand information on food packaging?	3 (2.8)	37 (33.9)	56 (51.4)	13 (11.9)	0 (0)	27 (23.9)	58 (51.3)	28 (24.8)
11	CORE-HL43	… to judge which everyday behaviour is related to your health?	0 (0)	12 (11.0)	74 (67.9)	23 (21.1)	0 (0)	9 (8.0)	69 (61.1)	35 (31.0)
12	CORE-HL44	… to make decisions to improve your health?	2 (1.8)	19 (17.4)	69 (63.3)	19 (17.4)	0 (0)	15 (13.3)	60 (53.1)	38 (33.6)

CR= Cardiac rehabilitation; HL = health literacy.

There were no statistically significant differences in HL score across categories of sex, age, nor years of education beyond upper secondary school (Table 3) neither at pre-CR nor at post-CR. The ANOVA analysis showed a statistically significant difference in HL scores across the three types of rehabilitation program, both at pre-CR (*F* = 3.39, *p* = 0.037) and post-CR (*F* = 5.75, *p* = 0.004). Post-hoc comparisons using the Tukey HSD test, displayed a statistically significant difference between the HL mean score of the patients offered 12 weeks outpatient rehabilitation (*M* = 36.79, *SD* = 3.78) compared to the mean score of those who were offered one-week residential rehabilitation (*M* = 34.26, *SD* = 4.35) at pre-CR. Similar results were found post-CR, with statistically significantly higher HL mean score for patients offered 12 weeks outpatient rehabilitation (*M* = 39.32, *SD* = 3.09) compared to those offered one-week residential rehabilitation (*M* = 35.77, *SD* = 4.94). The HL mean score of patients offered four weeks residential rehabilitation did not differ significantly from either patients offered one week residential rehabilitation or those offered twelve weeks outpatient rehabilitation.

**Table 3:** Health literacy mean scores across levels of sociodemographic variables and type of rehabilitation program.

	Pre-CR			Post-CR		
	*n* (%)	HL mean (sd)	*P* value	*n* (%)	HL mean (sd)	*P* value
Sex	*n* (%)					
**male**	84 (78)	35.36 (4.40)	0.555	84 (78)	37.40 (4.58)	0.828
**female**	25 (22)	34.76 (4.54)		25 (22)	37.64 (5.28)	
Age						
40–58	50 (46)	34.44 (3.78)	0.089	54 (48)	37.50 (4.09)	0.992
59–77	59 (54)	35.88 (4.83)		59 (52)	37.51 (5.18)	
Education	*n* (%)					
**0 years**	35 (32)	34.51 (4.76)	0.368	36 (32)	36.39 (5.30)	0.104
**1**–**3.5 years**	30 (28)	35.03 (4.47)		32 (28)	37.25 (4.61)	
**4**–**12 years**	44 (40)	35.91 (4.09)		45 (40)	38.58 (4.00)	
Type of rehabilitation	*n* (%)					
**1 week residential**	35 (32)	34.26 (4.35)	0.037	35 (31)	35.77 (4.94)	0.004
**4 weeks residential**	40 (37)	34.73 (4.70)		40 (35)	37.30 (5.14)	
**12 weeks outpatients**	34 (31)	36.79 (3.78)		38 (34)	39.32 (3.09)	

CR= Cardiac rehabilitation; HL = health literacy, sd = standard deviation; Education is measured as years beyond upper secondary school.

A statistically significant increase in HL score was found from the pre-CR (*M* = 35.22, *SD* = 4.42) to the post-CR (*M* = 37.46, *SD* = 4.73). The mean change in HL score from pre-to post CR was 2.24 ± 3.68 (*p* < 0.001), and the eta squared statistic (0.27) indicated large effect size. Exploring the change in HL score across levels of sociodemographic variables (Table 4) demonstrated no statistically significant differences.

**Table 4:** Change in HL scores across levels of sociodemographic variables and type of rehabilitation program.

	Change in HL score		
	*n* (%)	mean (sd)	*P* value
Sex	*n* (%)		
**male**	84 (78)	2.05 (3.82)	0.323
**female**	25 (22)	2.88 (3.18)	
Age			
**40**–**58**	50 (46)	2.96 (3.51)	0.059
**59**–**77**	59 (54)	1.63 (3.74)	
Education	*n* (%)		
**0 years**	35 (32)	1.80 (3.77)	0.583
**1**–**3.5 years**	30 (28)	2.13 (3.26)	
**4**–**12years**	44 (40)	2.66 (3.91)	
Type of rehabilitation	*n* (%)		
**1 week residential**	35 (32)	1.51 (3.64)	0.372
**4 weeks residential**	40 (37)	2.58 (4.08)	
**12 weeks outpatient**	34 (31)	2.59 (3.19)	

HL:health literacy, sd:standard deviation, Education is measured as years beyond upper secondary school.

## 4 Discussion

To our knowledge, the present study is the first study describing and evaluating changes in HL, measured with HLS-Q12 in patients attending a CR-program. In the present study, 39.4 % found it difficult or very difficult pre-CR to judge advantages and disadvantages of different treatment options. This is in line with former findings from both national (n = 2999) and international (n = 42,455) normative data [26,27], where respectively 44 % [26] and 43 % [27] found this difficult or very difficult. These findings are important as this item may reflect the patient’s capability for shared decision making, which is a key factor to enable user involvement as emphasized in the current guidelines [1]. Shared decision making has been defined as “an approach where clinicians and patients make decisions together using the best available evidence” [[28], p. 971]. In such a process, patients are encouraged to think about treatment or management options and the likely advantages and disadvantages, so that they are capable to communicate their preferences and help select the best option of action for them [28]. Thus, shared decision making respects patient autonomy as well as promotes patient engagement [28]. Shared decision making has been emphasized in a recently published statement on how to optimize adherence to a guideline-directed medical therapy in the secondary prevention of CVD [4]. Based on our findings and the aforementioned population surveys [26,27], it is however a timely question whether the patients are capable of shared decision making when entering CR.

At pre-CR, 39.5 % of the patients found judging whether the information on health risks in the mass media was reliable (item 7) as difficult or very difficult, and 40.4 % found deciding how they could protect themselves from illness using advice from family and friends (item 8) as difficult or very difficult. This finding is also in line with population surveys investigating HL [26,27]. We believe that these items, as well as item 3 (to judge the advantages and disadvantages of different treatment options), reflects knowledge at a higher cognitive level than most of the other items in HLS-Q12. According to Blooms taxonomy [29] the items of which our sample found most difficult assumes metacognitive knowledge as it assume awareness and knowledge of one's own cognition to be able to evaluate and judge health information and claims. This level of knowledge and the cognitive skills needed can be understood as critical HL, which is considered as more advanced cognitive abilities that enable people to critically assess health information and using information to exert greater control over life situations [[30], p. 264]. In order to achieve such a level of knowledge and cognitive skills, we believe that it places rather high demands on the pedagogic used in CR, both in group-based education as well as in individual consultations. Considering that mostly healthcare providers without any pedagogic background has been responsible for teaching and individual consultations at the CR-programs of which our sample are recruited from, it is gratifying that the most challenging HL-items at pre-CR improved during CR.

A reasonable and likely cause of the change observed in HL during CR in our study can be related to the content of the CR-programs. The core components of CR are intended to increase the patient’s knowledge so that they are better capable to manage their disease and to change and adhere to a healthy behaviour [1,31]. Therefore, it is clearly positive that patients consider themselves better equipped to take care of their own health post-CR. However, whether the knowledge gained in CR, which we believe are reflected in the statistically significant increase in HL, are at such a level that it improves adherence to healthy behaviour in long-term, remains unanswered.

Actions towards five dimensions are proposed to optimize therapy adherence in CVD [4]. These dimensions include the patient, the healthcare provider, the therapy, the healthcare system and the disease itself [4]. Comprehensive CR programs targets the proposed actions against the patient dimension [4], where increasing the patients HL is one of the three proposed actions. Since patients achieve increased HL as demonstrated in the present study, and the effects of comprehensive CR regarding CVD risk factors are well demonstrated [1], it seems that actions towards the patient are well taken care of. However, considering the fact that adherence to healthy behaviour adapted and/or initiated in CR are challenging in the long-term, it may be that to little action is directed towards the other dimensions proposed by the European Association of Preventive Cardiology [4]. In particular, actions towards the healthcare provider and the healthcare system seems to be warranted to improve adherence in the long-term [11].

The patients’ HL in secondary prevention may be a key to meet the challenge related to adherence to healthy behaviour [4,13]. Therefore, assessment of HL pre-CR and considering this assessment when tailoring the CR program for each individual, may be crucial. For this, a valid and easy to use tool are needed. Based on our experience, HLS-Q12 is easy to use in terms of time spent and the patients' understanding of the questions. However, how to interpret the individual scores to tailor the CR-program has not been investigated. Interestingly, we found that patients attending the 1-week residential CR-program had statistically significant lower levels of HL compared to those attending the 12-weeks outpatient CR-program. Not surprisingly, due to the length of the CR-program, patients in the 1-week residential CR-program also have the lowest increase in HL during CR. Based on our study, level of HL could serve as a guidance for which CR-program needed, and in addition, help healthcare providers to tailor the CR even better than today.

Despite the increased focus on the importance of HL in patients with noncommunicable diseases, evaluation of CR regarding HL is sparse. Consequently, there are few studies that we can compare our results against. In a study aimed to describe HL among CR attendees in Australia, a statistically significant increase in HL was found from CR-entry to post-CR (n = 38) [32]. Health literacy was evaluated with the Health Literacy questionnaire (HLQ) [33], and the statistically significant increase in HL was primarily related to scale 2 “having sufficient information to manage my health” [32]. However, the primary aim of the study by Beauchamp and colleagues [32] was to describe HL in patients attending CR. Since we used HLS-Q12 in the present study, comparisons of HL of the samples are not possible. It will be of great interest when the results from the ENHEARTEN study [34] are published as a comparison of the samples across the borders can be discussed. In their planned study, the relationship between HL and different health outcomes will be investigated by using HLS-Q12 in a cohort of 450 patients following their first myocardial infarction [34].

The use of technology has been proposed to meet the challenges related to adherence to healthy behaviour post-CR [35]. In 2020, we demonstrated that individual follow-up with an app for one-year post-CR, significantly improved adherence to healthy behaviour compared to usual care [17]. Whether adherence to healthy behaviour in this sample associates with level of HL may be difficult to assess due to a relatively small sample for this purpose. However, whether level of HL change over time (1- and 5-years post-CR) and whether adherence to healthy behaviour 4-year post intervention is still favourable for the intervention group that received individual follow-up with an app for one-year post-CR, will be investigated in upcoming studies.

## 5 Strengths and limitations

One of the strengths of this study is that there were no dropouts from pre-to post-CR. In addition, we were able to study the change in HL across three different types of CR. By including patients from different CR-programs which differs in terms of whether they are residential or outpatient programs as well as duration, we find the sample included representative for the European CR population [36].

Analysis of fit to the Rasch model is based on chi-square statistics which is dependent of sample size [37]. As the sample size is relatively low, there might be weaknesses of the HLS-Q12 instrument when it comes to fit statistics and DIF that are unrevealed in this study. However, considering this study having a pre-post-test design, the sample is sufficient [38].

## 6 Conclusion

Participation in CR statistically significantly improves HL. There were no statistically significant differences in change of HL in the CR population regarding age, sex, educational level, or type of CR program attended. Judging health information was found as the most difficult aspect of HL. This should be considered by healthcare providers in secondary prevention to improve health outcomes.

## CRediT authorship contribution statement

**Pernille Lunde:** Writing – review & editing, Writing – original draft, Visualization, Project administration, Methodology, Investigation, Funding acquisition, Formal analysis, Data curation, Conceptualization. **Jostein Grimsmo:** Writing – review & editing, Project administration, Methodology, Investigation, Data curation, Conceptualization. **Birgitta Blakstad Nilsson:** Writing – review & editing, Supervision. **Asta Bye:** Writing – review & editing, Supervision, Funding acquisition. **Hanne Søberg Finbråten:** Writing – review & editing, Writing – original draft, Methodology, Formal analysis, Conceptualization.

## Declaration of competing interest

The authors report no relationships that could be construed as a conflict of interest.

## References

[1] Ambrosetti M., Abreu A., Corrà U., Davos C.H., Hansen D., Frederix I. (2020). Secondary prevention through comprehensive cardiovascular rehabilitation: from knowledge to implementation. 2020 update. A position paper from the Secondary Prevention and Rehabilitation Section of the European Association of Preventive Cardiology. European journal of preventive cardiology.

[2] World Health Organization (2003). https://apps.who.int/iris/handle/10665/42682.

[3] Kotseva K., Wood D., De Bacquer D., De Backer G., Rydén L., Jennings C. (2016). EUROASPIRE IV: a European Society of Cardiology survey on the lifestyle, risk factor and therapeutic management of coronary patients from 24 European countries. European Journal of Preventive Cardiology.

[4] Pedretti R.F., Hansen D., Ambrosetti M., Back M., Berger T., Ferreira M.C. (2023). How to optimize the adherence to a guideline-directed medical therapy in the secondary prevention of cardiovascular diseases: a clinical consensus statement from the European Association of Preventive Cardiology. European journal of preventive cardiology.

[5] Sørensen K., Van den Broucke S., Fullam J., Doyle G., Pelikan J., Slonska Z. (2012). Health literacy and public health: a systematic review and integration of definitions and models. BMC Publ. Health.

[6] Machado B., Fernandes A., Cruzeiro S., Jesus R., Araújo N., Araújo I. (2019). Cardiac rehabilitation program and health literacy levels: a cross‐sectional, descriptive study. Nurs. Health Sci..

[7] Tschaftary A., Hess N., Hiltner S., Oertelt-Prigione S. (2018). The association between sex, age and health literacy and the uptake of cardiovascular prevention: a cross-sectional analysis in a primary care setting. J. Publ. Health.

[8] Williams M.V., Baker D.W., Parker R.M., Nurss J.R. (1998). Relationship of functional health literacy to patients' knowledge of their chronic disease: a study of patients with hypertension and diabetes. Arch. Intern. Med..

[9] Macabasco-O’Connell A., DeWalt D.A., Broucksou K.A., Hawk V., Baker D.W., Schillinger D. (2011). Relationship between literacy, knowledge, self-care behaviors, and heart failure-related quality of life among patients with heart failure. J. Gen. Intern. Med..

[10] Mayberry L.S., Schildcrout J.S., Wallston K.A., Goggins K., Mixon A.S., Rothman R.L. (2018). Mayo Clinic Proceedings.

[11] Beauchamp A., Talevski J., Niebauer J., Gutenberg J., Kefalianos E., Mayr B. (2022). Health literacy interventions for secondary prevention of coronary artery disease: a scoping review. Open Heart.

[12] Visseren F.L., Mach F., Smulders Y.M., Carballo D., Koskinas K.C., Bäck M. (2022). 2021 ESC Guidelines on cardiovascular disease prevention in clinical practice: developed by the Task Force for cardiovascular disease prevention in clinical practice with representatives of the European Society of Cardiology and 12 medical societies with the special contribution of the European Association of Preventive Cardiology (EAPC). European journal of preventive cardiology.

[13] Brørs G., Dalen H., Allore H., Deaton C., Fridlund B., Osborne R.H. (2022). Health literacy and risk factors for coronary artery disease (from the CONCARDPCI study). Am. J. Cardiol..

[14] Nilsson B.B., Lunde P., Holm I. (2017). Implementation and evaluation of the Norwegian Ullevaal model as a cardiac rehabilitation model in primary care. Disabil. Rehabil..

[15] Bergum H., Sandven I., Abdelnoor M., Anderssen S.A., Grimsmo J., Rivrud D.E. (2022). Randomized trial of cardiovascular prevention in Norway combining an in-hospital lifestyle course with primary care follow-up: the Hjerteløftet study. European Journal of Preventive Cardiology.

[16] Moholdt T., Bekken Vold M., Grimsmo J., Slørdahl S.A., Wisløff U. (2012). Home-based aerobic interval training improves peak oxygen uptake equal to residential cardiac rehabilitation: a randomized, controlled trial. PLoS One.

[17] Lunde P., Bye A., Bergland A., Grimsmo J., Jarstad E., Nilsson B.B. (2020). Long-term follow-up with a smartphone application improves exercise capacity post cardiac rehabilitation: a randomized controlled trial. European Journal of Preventive Cardiology.

[18] Finbråten H.S., Wilde-Larsson B., Nordström G., Pettersen K.S., Trollvik A., Guttersrud Ø. (2018). Establishing the HLS-Q12 short version of the European Health Literacy Survey Questionnaire: latent trait analyses applying Rasch modelling and confirmatory factor analysis. BMC Health Serv. Res..

[19] Sørensen K., Van den Broucke S., Pelikan J.M., Fullam J., Doyle G., Slonska Z. (2013). Measuring health literacy in populations: illuminating the design and development process of the European Health Literacy Survey Questionnaire (HLS-EU-Q). BMC Publ. Health.

[20] Finbråten H.S., Pettersen K.S., Wilde‐Larsson B., Nordström G., Trollvik A., Guttersrud Ø. (2017). Validating the European Health Literacy Survey Questionnaire in people with type 2 diabetes: latent trait analyses applying multidimensional Rasch modelling and confirmatory factor analysis. J. Adv. Nurs..

[21] Masters G.N. (1982). A Rasch model for partial credit scoring. Psychometrika.

[22] Rasch G. (1960).

[23] Andrich D., Marais I. (2019). A course in Rasch measurement theory. Measuring in the educational, social and health sciences.

[24] Tennant A., Conaghan P.G. (2007). The Rasch measurement model in rheumatology: what is it and why use it? When should it be applied, and what should one look for in a Rasch paper?. Arthritis Care Res..

[25] Andrich D., Sheridan B. (2019). RUMM2030Plus. Rumm Laboratory Pty Ltd: Duncraig, Australia.

[26] Le C., Finbråten H.S., Pettersen K.S., Joranger P., Guttersrud Ø. (2021). The International Health Literacy Population Survey 2019–2021 (HLS19)–Et Samarbeidsprosjekt Med Nettverket M-POHL Tilknyttet WHO-EHII.

[27] M-POHL THCotWAN (2021).

[28] Elwyn G., Laitner S., Coulter A., Walker E., Watson P., Thomson R. (2010). Implementing shared decision making in the NHS. BMJ.

[29] Krathwohl D.R. (2002). A revision of Bloom's taxonomy: an overview. Theory Into Pract..

[30] Nutbeam D. (2000). Health literacy as a public health goal: a challenge for contemporary health education and communication strategies into the 21st century. Health Promot. Int..

[31] Piepoli M.F., Corra U., Adamopoulos S., Benzer W., Bjarnason-Wehrens B., Cupples M. (2014). Secondary prevention in the clinical management of patients with cardiovascular diseases. Core components, standards and outcome measures for referral and delivery: a policy statement from the cardiac rehabilitation section of the European Association for Cardiovascular Prevention & Rehabilitation. Endorsed by the Committee for Practice Guidelines of the European Society of Cardiology. Eur J Prev Cardiol.

[32] Beauchamp A., Sheppard R., Wise F., Jackson A. (2020). Health literacy of patients attending cardiac rehabilitation. J. Cardpulm. Rehabil. Prev..

[33] Osborne R.H., Batterham R.W., Elsworth G.R., Hawkins M., Buchbinder R. (2013). The grounded psychometric development and initial validation of the Health Literacy Questionnaire (HLQ). BMC Publ. Health.

[34] Beauchamp A., Talevski J., Nicholls S.J., Shee A.W., Martin C., Van Gaal W. (2022). Health literacy and long-term health outcomes following myocardial infarction: protocol for a multicentre, prospective cohort study (ENHEARTEN study). BMJ Open.

[35] Piepoli M.F., Hoes A.W., Agewall S., Albus C., Brotons C., Catapano A.L. (2016). European Guidelines on cardiovascular disease prevention in clinical practice. Atherosclerosis.

[36] Ruivo J., Moholdt T., Abreu A. (2023). Overview of Cardiac Rehabilitation following post-acute myocardial infarction in European Society of Cardiology member countries. European Journal of Preventive Cardiology.

[37] Lantz B. (2013). The large sample size fallacy. Scand. J. Caring Sci..

[38] Carter R.E., Lubinsky J., Domholdt E. (2011).

